# Surgical management of retinal detachment and macular holes secondary to ocular toxoplasmosis: a systematic review and meta-analysis

**DOI:** 10.1186/s40942-024-00540-w

**Published:** 2024-02-29

**Authors:** Dillan Cunha Amaral, Mark Lane, Eduardo Henrique Cassins Aguiar, Guilherme Nunes Marques, Luiza Visentin Cavassani, Márcio Penha Morterá Rodrigues, Milton Ruiz Alves, José Eduardo Ferreira Manso, Mário Luiz Ribeiro Monteiro, Ricardo Noguera Louzada

**Affiliations:** 1 Faculdade de Medicina, Universidade Federal do Rio de Janeiro, Rio de Janeiro, Brazil; 2 Moorfields Eye Hospital, London, UK; 3 Faculdade de Medicina, Centro Universitário Max Planck, Indaiatuba, Brazil; 4 Faculdade de Medicina, Universidade de São Paulo, São Paulo, Brazil; 5 Instituto de Olhos São Sebastião, Largo Do Machado 54, 1208, Rio de Janeiro, 22221-020 Brazil

**Keywords:** Ocular toxoplasmosis, Retinal detachment, Meta-analysis, Retinal break, Macular hole

## Abstract

**Background:**

Toxoplasma gondii causes ocular toxoplasmosis (OT), involving inflammation, scarring, and retinal complications. The OT complications were retinal detachment (RD), and retinal breakage (RB). Surgical interventions like scleral buckling (SB) and vitrectomy are common. Limited understanding exists of the safety and efficacy of surgical management of RD/RB secondary to OT. Another complication is toxoplasmosis-related macular holes (tMH), with sparse evidence on surgical outcomes. This meta-analysis aims to clarify clinical characteristics, and surgical results, and enhance understanding of RD, RB, and MH secondary to OT.

**Methods:**

PubMed, Cochrane, Embase and Web of Science database were queried for retrospective studies, case series and case reports that provided information on RD, RB and MH associated with OT and reported the outcomes of: (1) Retinal reattachment of RD/RB and tMH closure; (2) Best-corrected visual acuity (BCVA) improvement; and (3) Complications. Heterogeneity was examined with I^2^ statistics. A random-effects model was used for outcomes with high heterogeneity. Statistical analysis was performed using the software R (version 4.2.3, R Foundation for Statistical Computing, Vienna, Austria).

**Results:**

Fourteen final studies, comprising a total of 96 patients were analyzed, 81 with RD or RB and 15 with tMH. Overall, surgical management was associated with several advantages: a high rate of retinal reattachment of RD/RB of 97% (95% Confidence Interval [CI] 92–100%; I^2^ = 0%), retinal reattachment of just RD of 96% (95% CI 89–100%; I^2^ = 30%) and tMH closure 97% (95% CI 87–100; I^2^ = 12%). There were significant differences in BCVA after surgeries in studies of RD/RB (MD 0.60; 95% CI 0.35–0.65; I^2^ = 20%) and MH (MD 0.67; 95% CI 0.50–0.84; I^2^ = 0%). The overall complication rate associated with surgical procedures in RD/RB secondary to OT was confirmed to be 25%.

**Conclusions:**

The systematic review and meta-analysis showed that the treatment approaches currently in use are effective, with a remarkable rate of retinal reattachment of RD/RB, tMH closure, and substantial improvements in visual acuity. More randomized, long-term studies on disease and surgical factors can provide valuable insights into their impact on anatomical and visual outcomes.

## Introduction

*Toxoplasma gondii* is frequently characterized as one of the most prolific parasites; it hosts a wide range of organisms that encompasses humans, as well as domesticated and wild warm-blooded animals [1]. The clinical sequelae ensuing from an infection with *T. gondii* are medically designated toxoplasmosis. Specifically, when ocular structures are involved, the condition is labeled ocular toxoplasmosis (OT). The modes of infection encompass congenital transmission as well as postnatal acquisition, with empirical research substantiating a predominantly postnatal origin [2]. During the acute phase of infection, the tachyzoites of the parasite infiltrate ocular tissues, precipitating an inflammatory cascade that manifests as inflammation, necrosis, fibrotic scarring, retinal and choroidal atrophy (termed retinochoroiditis), and inflammation affecting the optic nerve head (referred to as papillitis) and the uvea (designated uveitis) [3]. Chronic ocular toxoplasmosis is typified by the presence of parasitic cysts within the retina, ganglion cells, and Muller cells [4].

Some individuals with OT may exhibit retinal lesions [5, 6], including retinal detachment (RD), which refers to the detachment of the anterior sensory retinal layers from the retinal pigment epithelium (RPE), leading to the accumulation of subretinal fluid within the interstitial space separating the retina from the RPE [7]. Notably, in patients marked by substantial scarring and inflammation involving the peripheral retinal regions, the risk of retinal detachment is significantly heightened. The presence of scarring and inflammation resulting from ocular toxoplasmosis can exert tractional forces upon the retina, thus accentuating the likelihood of RD and retinal breakage (RB) [8]. The management of RD typically involves surgical techniques such as scleral buckling (SB), pars plana vitrectomy (PPV), and pneumatic retinopexy (PR) [9, 10]. However, the prevalence and visual consequences of RD in patients with OT have not been fully characterized. There is a significant gap in our understanding of the anatomical and functional outcomes of patients with toxoplasmic RD. Moreover, factors related to the disease and its treatment that influence the incidence of RD and poor visual outcomes are not well understood in the literature.

Moreover, another potential retinal complication to consider is the development of a toxoplasmosis-related macular hole (tMH). While relatively rare, there is a belief that inflammatory conditions may instigate the migration and proliferation of the RPE within the retina. This process can result in the shrinkage of the retina and the generation of tangential traction on the macula, potentially contributing to the development of a tMH. However, it is crucial to note that there is a scarcity of evidence on this aspect, with only a limited number of studies specifically investigating surgical outcomes related to tMH.

To enhance our understanding of the pathogenesis and management of toxoplasmosis-related retinal lesions, this meta-analysis aimed to comprehensively assess the clinical characteristics of individuals with RD, RB and MH associated with ocular toxoplasmosis while also examining surgical treatment outcomes for these conditions.

## Methods

### Protocol, search strategy and data extraction

We systematically searched the PubMed, Cochrane Library, Embase, and Web of Science databases. This study was registered in the International Prospective Register of Systematic Reviews (PROSPERO; CRD42023482369). Our search strategy was carefully crafted to conduct a thorough investigation of the topic utilizing a comprehensive combination of relevant keywords. The specific keywords employed in our search included: "Toxoplasmosis", "Toxoplasmic", "Ocular Toxoplasmoses", "Ocular Toxoplasmosis", “Detachment, Retinal", "Detachments, Retinal", "Retinal Detachments", "Retinal Pigment Epithelial Detachment", "Retinal Perforation", "Holes, Retinal", "Macular Hole", "Macular Holes", "Retinal Break", "Retinal Breaks", "Retinal Dialyses", "Retinal Hole", "Retinal Holes", "Retinal Perforation", "Retinal Tear", "Retinal Tears", "Retinal Detachment". This meticulous approach ensured that we obtained the most pertinent and reliable information, empowering us to present a well-founded and in-depth analysis of the subject matter. Two authors (L.C. and G.M.) independently extracted the data following predefined search criteria.

### Eligibility criteria

The inclusion criteria of this study were as follows: (1) Participants: individuals (> 18 years) with RD and/or RB associated with ocular toxoplasmosis and individuals (> 18 years) with tMH (2) Intervention: surgical or clinical therapy; (3) At least one or more clinical outcomes: retinal reattachment, tMH closure, recurrence of RD or RB, development of RD or RB less than 2 years after Retinochoroiditis, best-corrected visual acuity (BCVA) improvement and complications; (4) Type of study: retrospective observational studies, case series, and case reports. The exclusion criteria were as follows: (1) animal studies; (2) abstracts, editorials, letters, and conference proceedings without efficient data; (3) research papers that did not provide clear data. This exclusion was implemented to ensure that only high-quality studies were included in the analysis.

### Statistical analysis

This systematic review and meta-analysis were performed per the Cochrane Collaboration and the Preferred Reporting Items for Systematic Reviews and Meta-Analysis (PRISMA) statement guidelines [11]. Relative risk (RR) with 95% confidence intervals (CIs) were used to compare outcome treatment effects. Continuous outcomes were compared with mean difference (MD). I^2^ statistics were used to assess for heterogeneity; When P < 0.1 for the Q test and I^2^ > 50%, which was considered as substantial heterogeneity, the random-effect model was used; otherwise, the fixed-effect model was performed. Statistical analysis was performed using the software R (version 4.2.3, R Foundation for Statistical Computing, Vienna, Austria).

### Individual patient data meta-analysis

In our comprehensive systematic review and meta-analysis, we categorized studies based on the level of granularity in the patient data they supplied. The individual patient data meta-analysis (IPDMA) group comprised case reports studies that offered individual-level patient data. For our analytical purposes, the IPDMA studies were amalgamated into two distinct groups. The first group, termed IPDMA-RD, encompassed case reports featuring patients with RD and/or RB. The second group, designated IPDMA-MH, included case reports involving patients with tMH.

## Results

### Study selection

**Fig. 1:**
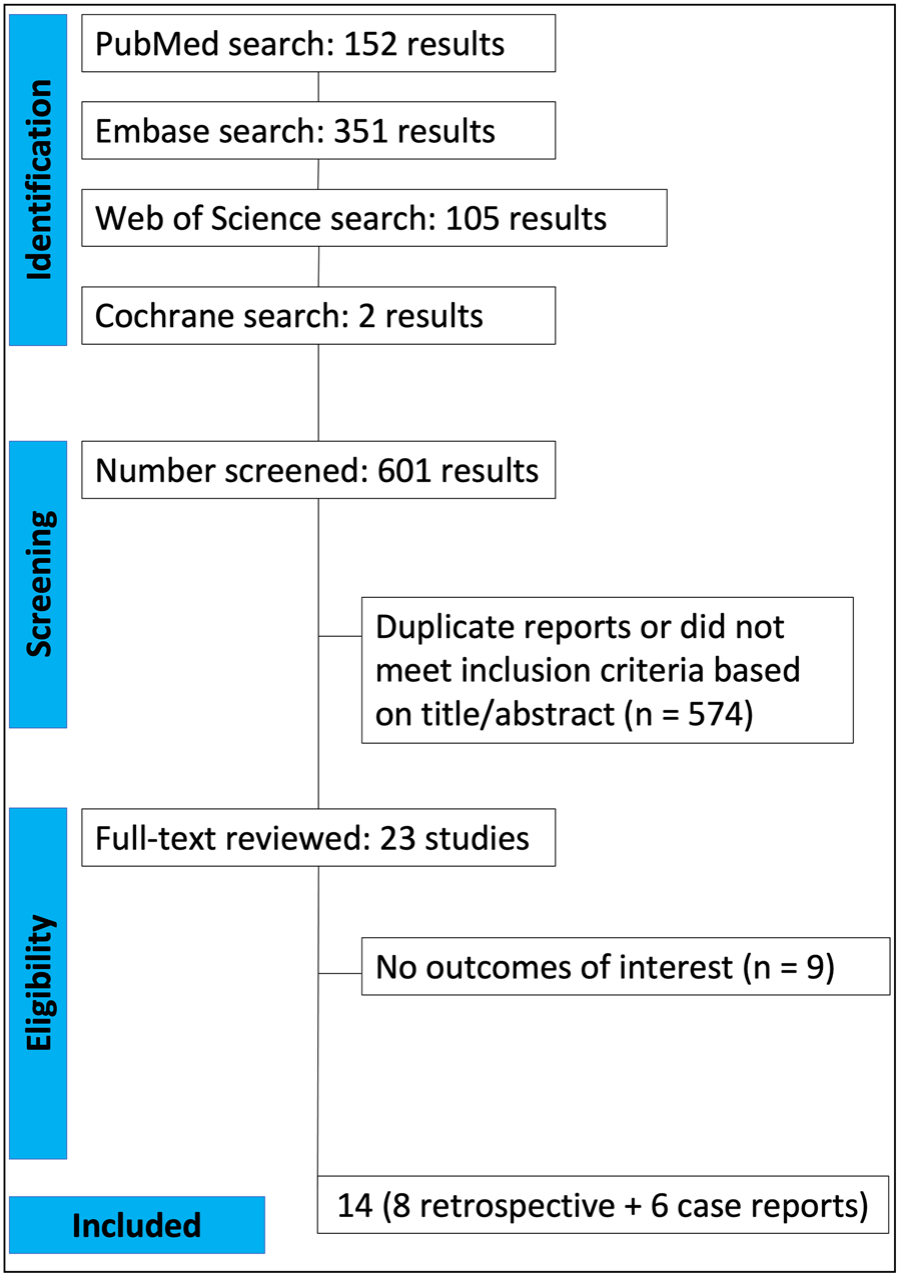
PRISMA flow diagram of study screening and selection

We found 610 articles, 152 in PubMed, 351 in Embase, 105 in the Web of Science, and 2 in the Cochrane Library. A total of 390 nonduplicate citations were screened, and after a thorough review, 23 articles were selected after the abstracts were read for a full-text review. Next, 9 articles were excluded after full-text screening and data extraction. Finally, 14 studies (8 retrospective studies [12–19] + 6 case reports [20–25]) were included in the final analysis. This search is described in Fig. 1.

In this analysis, a total of 96 patients were analyzed, 81 with RD or RB and 15 with MH. The baseline characteristics of the RD or RB studies are shown in Table 1, while Table 2 displays the tMH studies.

**Table 1:** Baseline characteristics of the included patients with RD or RB

Study (year)	Design	Number of patients	M/F ratio	Mean age	Associated Pathologies	Follow-up	Worsening in visual acuity	Number of RD or RB	Retinal Reattachment	Type of RD or RB	Intervention	Clinical Treatment
Moreira (2018)	Retrospective	22	13/9	28.5	Proliferative Vitreoretinopathy (5/22);	64.9 months	2	22	20/22	RRD (22/22)	RSB (4/22); PPV + GAS(1/22); PPV + SO(4/22); RSB + GAS (3/10); RSB + PPV + SO (10/22)	NA
Lucena (2009)	Retrospective	10	3/7	38	NA	NA	3	10	10/10	RD + RR (8/10); RRP (retinal rupture) (2/10)	Retinopexy + PPV (2/10); PPV (4/10); Retinopexy (2/10); Laser (2/10)	NA
Kianersi (2012)	Retrospective	5	1/4	27	NA	1 month	5	5	5/5	RRD (3/5) + TRD (1/5) + Unknow URD (1/5)	PPV + lensectomy(1/5); SB + Crypexia (1/5); SB (2/5); PPV + SO(1/5)	Antiparasitics with corticosteroids (oral and topical), mydriatic agents (4/5); None (1/5)
Faridi (2015)	Retrospective	28	14/14	40	NA	22.5 months	3	4	4/4	RRD (4/4)	PPV + PFO + MP + AFX + EL + SO—> PPV/ROSO/PPL/MP/EL/retinectomy/SB/C3F8—> PPV/MP/EL/PFO/300° retinectomy/AFX/long-acting SO tamponade (1/4); PPV/AFX/EL/SF6—> PPV/EL/AFX/SB/SF6—> PPV/retinectomy/PFO/AFX/EL/SO/CE IOL/PPV/ROSO—> PPV/270° retinectomy/AFX/EL/ long-acting SO tamponade—> PPV/ROSO/MP; PPV/AFX/EL/MP/SO (1/4); Laser retinopexy (1/4)	Oral Antibiotic therapy [sulfamethoxazole/trimethoprim (9/28), triple therapy: sulfadiazine, pyrimethamine, and folinic acid, (6/28), clindamycin (3/28), or azithromycin (1/28), unknown (9/28)]; Intravitreal Therapy [intravitreal clyndamicin and dexamethasone (2/4), intravitreal clyndamicin and triamcinolone (1/4), intravitreal triamcinolone (1/4)]
Driessen (2000)	Retrospective	150	NA	29.5	Myopia + RB/RD (8/16); Myopia (35/134)	7 years	7	16	12/16	RD (8/16); RD + RB(1/16); RB (7/16)	SB + PPV + SO (1/16); SB (2/16); SB + lensectomy (1/16); Cryopexia + SB (1/16); PPV + SO + lensectomy (1/16); Cryocogulations 1/16 + Abstained (2/16); Laser coagulation (7/16)	Antiparasitics with corticosteroids (4/16); Antiparasitics (3/16); Corticosteroids (2/16); None (7/16)
Caplan (2023)	Retrospective	420	NA	40.9	Proliferative Vitreoretinopathy (4/13 RRD);	3 months	N/A	27 (16 analyzed)	14/14 (14 analyzed in last follow-up)	RRD (13/16); TRD (3/16)	SB + SO (2/14); PPV + SO (7/14); PPV + SB + SO (5/14)	NA
Adán (2009)	Retrospective	15	8/7	37.2	NA	41.4 months	0	8	8/8	RRD (6/8); RRD + TRD (2/8)	SB + PPV + SO (1/8); SB + PPV + delamination + GAS (1/8); SB + PPV + lensectomy + SO (2/8); SB + PPV + GAS (4/8)	Trimethoprim/sulfamethoxazole and oral prednisone at tapering doses for 30–40 days

**Study (year)**	**Design**	**M:F**	**Age**	**Associated Pathologies**	**Follow- up**	**Worsening in visual acuity**	**–**	**Anatomical closure**	**Type of RD or RB**	**Intervention**	**Clinical Treatment**
Scott (2018)	Case report	M	27	NA	5 months	YES	**–**	YES	Retinal tear + RRD	SB + PPV + GAS	Trimethoprim/sulfamethoxazole (160 mg/800 mg) twice daily and clindamycin 300 mg three times daily, as well as topical glucocorticoids and a mydriatic agent. One week later, he initiated systemic oral glucocorticoid therapy (prednisone 60 mg daily) with a scheduled taper
Erol (2021)	Case report	F	30	NA	2 months	YES	**–**	YES	MH + RD	PPV	Trimethoprim/sulfamethoxazole (160/800 mg) twice a day and clindamycin 300 mg four times a day. After three days, 32 mg/day methylprednisolone was added to oral treatment

*NA* not available,* PPV* pars plana vitrectomy,* RD* retinal detachment,* RRP* retinal rupture,* TRD* tractional retinal detachment,* URD* unknown retinal detachment,* RB* retinal break,* ILM* internal limiting membrane peeling,* ERM* epiretinal membrane peeling,* RSB* retinopexy with scleral buckling,* GAS* gas infusion,* SO* silicone oil infusion; SB scleral buckling,* RRD* rhegmatogenous retinal detachment; MH macular hole,* AFX* air–fluid exchange,* CE IOL* cataract extraction with intraocular lens placement,* EL* endolaser,* MP* membrane pee,* PFO* perfluorocarbon,* PPL* pars plana lensectomy,* ROSO* removal of silicone oil

**Table 2:** Baseline characteristics of the included patients with tMH

Study (year)	Design	Number of patients	M/F ratio	Mean age	Follow-up	Macular hole width	Macular hole closure	Type of MH	Intervention	Clinical Treatment for Ocular Toxoplasmosis	Clinical Treatment in Postoperative
Souza (2018)	Retrospective	11	5/6	33.2	6 months	165 ± 96 µm	11/11	tMH (11/11)	PPV + ILM + ERM (3/11); PPV + ILM (8/11)	NA	Postoperatively, all patients were prescribed moxifloxacin for 1 week and Pred Forte (prednisolone acetate 1%, Allergan) for 4 weeks, prophylactic TR treatment with trimethoprim/sulfamethoxazole (160/800 mg 3 times per week), and regularly followed up for at least 6 months

Study (year)	Design	M:F	Age	Follow-up	Macular hole width	Macular hole closure	Type of MH	Intervention	Clinical Treatment for Ocular Toxoplasmosis	Clinical Treatment in Postoperative
Erol (2021)	Case report	F	30	2 months	698 µm	Yes	FTMH	PPV	Trimethoprim/sulfamethoxazole (160/800 mg) twice a day and clindamycin 300 mg four times a day. After three days, 32 mg/day methylprednisolone was added to oral treatment	Drops containing antibiotics and steroids were applied for up to one month after surgery. Oral antibiotic treatment for toxoplasma was continued for six weeks
Tanaka (2014)	Case report	M	59	5 months	2852 μm horizontally, 2571 μm vertically in spectral-domain OCT	No	FTMH	PPV + ILM + GAS	Oral acetylspiramycin (1200 mg/day) for 8 weeks	NA
Arana (2012)	Case report	F	35	8 months	NA	Yes	FTMH	PPV + GAS	Prednisolone and cycloplegic drops and empirical oral treatment with sulphamethoxazole (800 mg)/trimethoprim (160 mg) twice a day were started. Oral prednisone at 60 mg/day with tapering doses was added 2 days later for 8 weeks	NA
Ikeda (2021)	Case report	M	49	7 months	500 μm	Yes	FTMH	PPV + ILM	400 mg of sulfamethoxazole and 80 mg of trimethoprim orally. The oral prednisolone was also started from 30 mg and was tapered in 1 month. The treatment was continued for 6 weeks	NA
Doshi (2020)	Case report	M	34	4 weeks	NA	Yes	FTMH	Clinical	Sulphamethoxazole and trimethoprim (800 mg/160 mg) with oral prednisolone (1 mg/kg). As the lesion was very close to the fovea, the patient also received a single dose of intravitreal clindamycin 1 mg/0.1 ml along with dexamethasone 400 microgm/0.1 ml	NA

M: male; F: female; MH: macular hole; tMH: toxoplasmosis-related macular hole; FTMH: full-thickness macular hole; PPV: pars plana vitrectomy; ILM: internal limiting membrane peeling; MH: macular hole; GAS: gas infusion

### Retinal reattachment

**Fig. 2:**
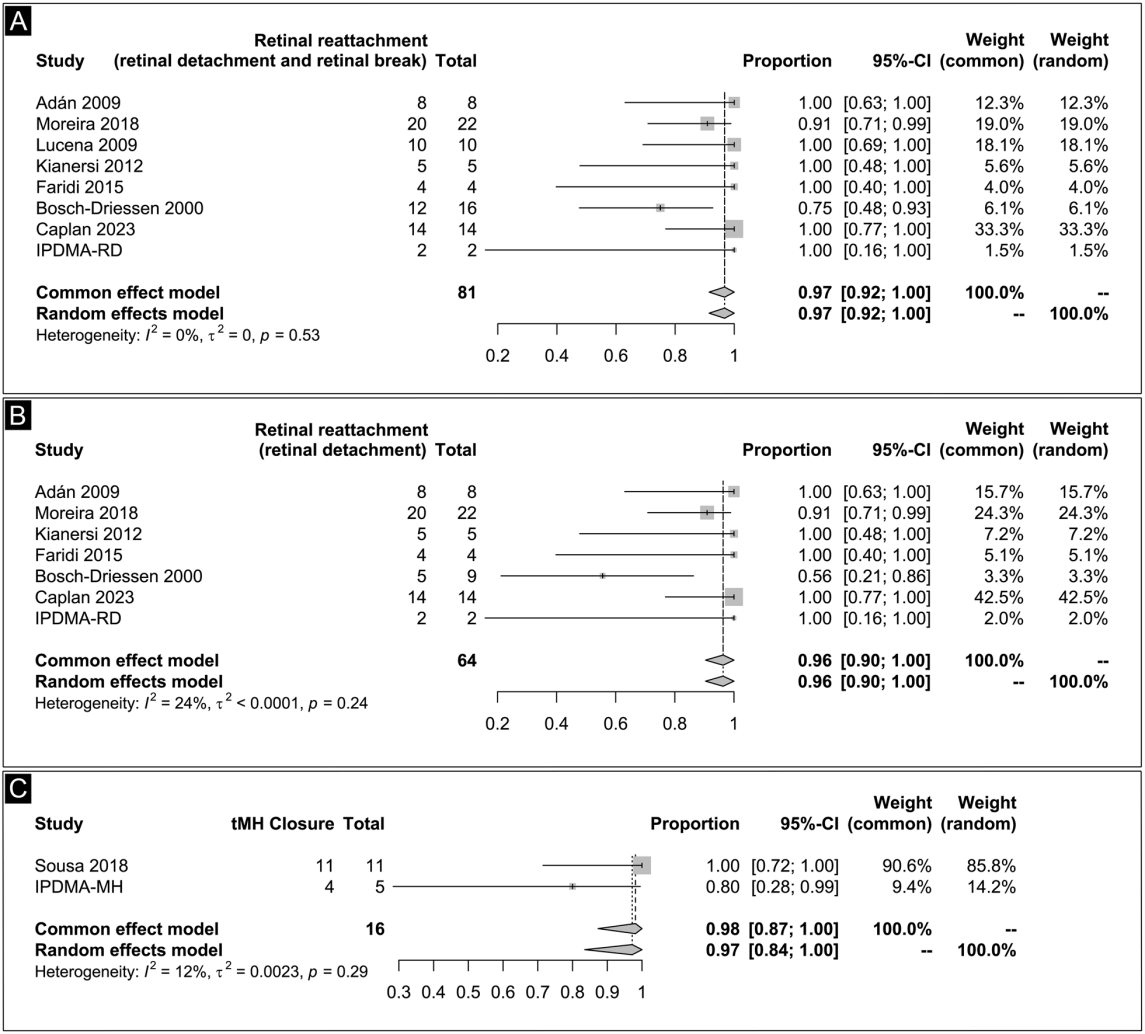
**A** Retinal reattachment of the retinal detachment and retinal breakage. **B** Retinal reattachment of retinal detachment forest plot. **C** Toxoplasmosis-related macular hole closure

The rate of retinal reattachment of RDs and RBs was reported in eight studies that included 81 eyes. We observed no methodological heterogeneity among the studies (P = 0.53, I^2^ = 0%). The pooled retinal reattachment rate was 97% (95% CI, 92 to 100%) in a common-effect model (Fig. 2A). Furthermore, the analysis of retinal reattachment in cases of RD were mildly heterogeneous (P = 0.24, I^2^ = 24%). The pooled retinal reattachment of RD was 96% (95 CI: 90 to 100%) with a common-effect model (Fig. 2B).

### Toxoplasmosis-related macular hole closure

The analysis of tMH closure revealed a rate of 98% (95% CI 87–100%), with a low level of heterogeneity (I^2^ = 12%; common-effect model), as shown in Fig. 2C.

### Development of retinal detachment or retinal breaks less than 1 year after retinochoroiditis

**Fig. 3:**
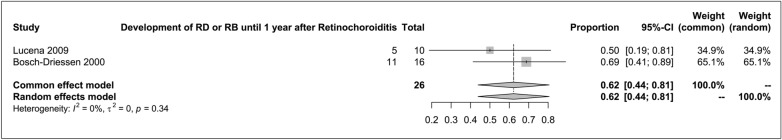
Development of retinal detachment or retinal breaks less than 1 year after retinochoroiditis forest plot

The rate of development of RD or RB in less than 1 year after retinochoroiditis was reported in two studies that included 26 eyes. We observed no methodological heterogeneity among the studies (P = 0.34, I^2^ = 0%). These pooled analysis rate was 62% (95% CI 44–81%) in a common-effect model (Fig. 3).

### Recurrence of retinal detachment

**Fig. 4:**
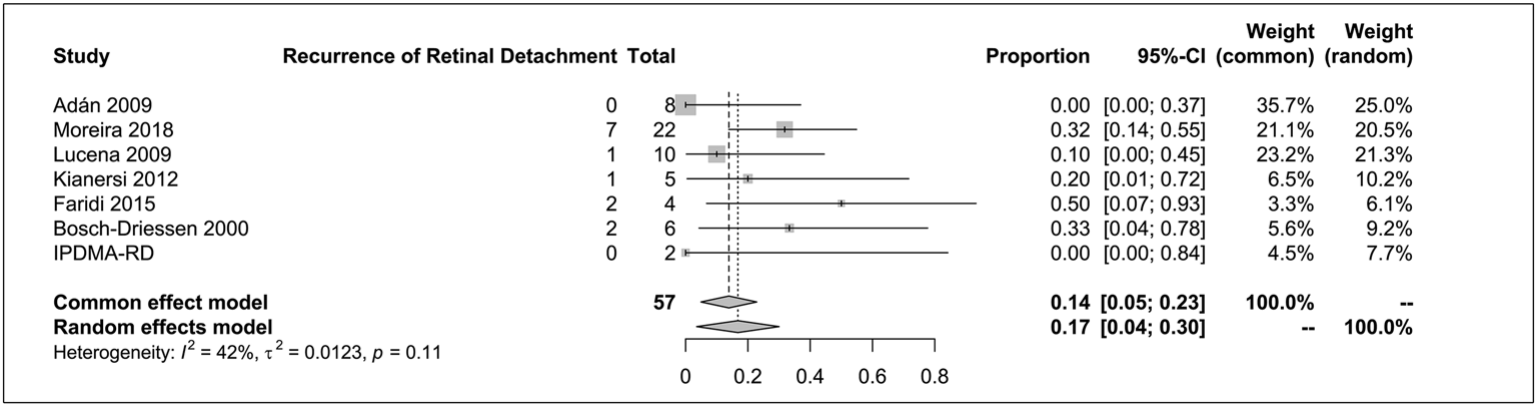
Recurrence of retinal detachment forest plot

The analysis of recurrence of RD revealed a rate of 14% (95% CI 5–23%), with a mild level of heterogeneity (I^2^ = 42%; common-effect model), as shown in Fig. 4.

### Best-corrected visual acuity

**Fig. 5:**
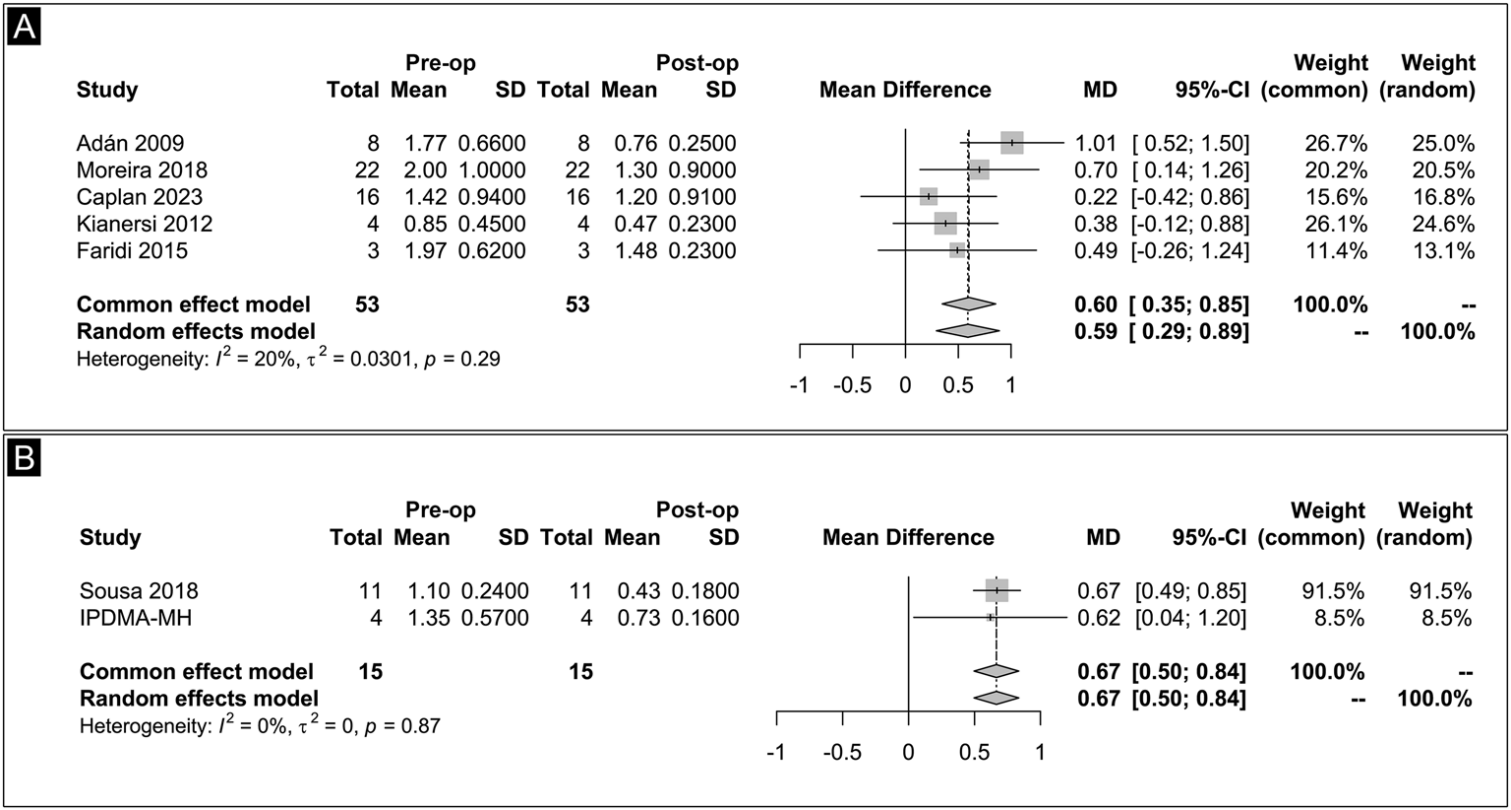
**A** Best-corrected visual acuity mean difference after RD and RB surgical management forest plot. **B** Best-corrected visual acuity mean difference after MH surgical management forest plot

The mean BCVA before and after RD or RB surgeries was documented in five studies, encompassing a total of 53 eyes. We observed a low level of heterogeneity among the studies (P = 0.29, I^2^ = 20%). Compared to the preoperative baseline, BCVA significantly improved after the surgical management (MD = 0.60; 95% CI 0.35–0.85; Fig. 5A).

The mean BCVA before and after MH surgeries encompassed a total of 15 eyes. We observed a low level of heterogeneity among the studies (P = 0.87, I^2^ = 0%). Compared to the preoperative baseline, BCVA significantly improved after surgical management (MD = 0.67; 95% CI 0.50–0.84; Fig. 5B).

### Complications

**Fig. 6:**
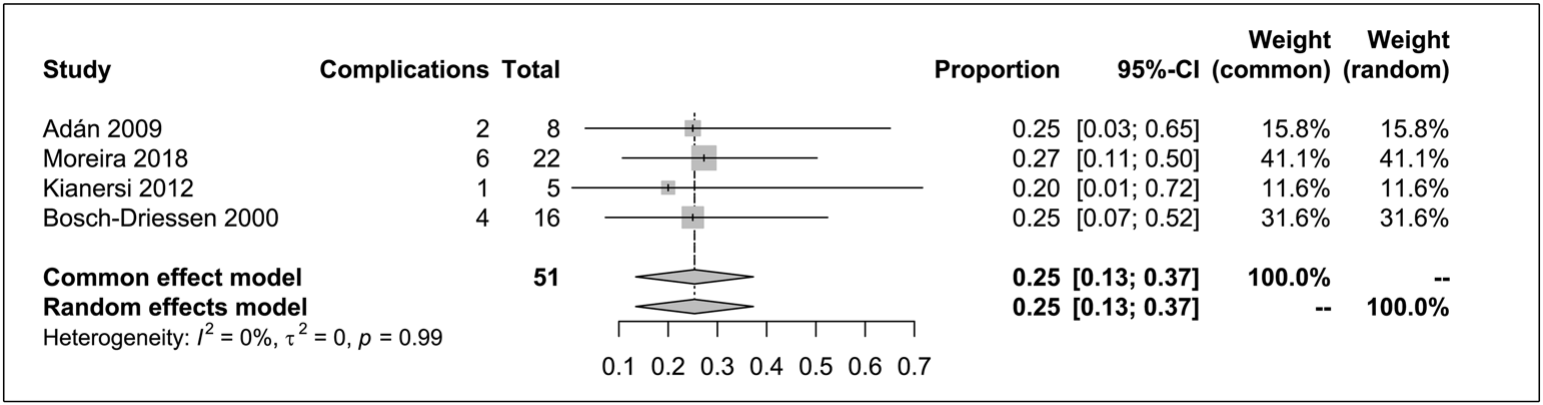
Complications associated with surgical management of retinal detachment secondary to ocular toxoplasmosis forest plot

The rate of complications was reported in four studies that included 51 eyes with RD or RB. We observed no methodological heterogeneity among the studies (P = 0.99, I^2^ = 0%). This pooled analysis rate was 25% (95% CI 13–37%) in a common-effect model (Fig. 6). The complications reported in RD or RB studies were glaucoma or elevated intraocular pressure, bulbar atrophy, hypotony, cataract, dislocation of lens and final enucleation. In the investigations of surgical interventions for MHs, the sole reported complication was the occurrence of a single cataract.

## Discussion

To our knowledge, this meta-analysis represents the inaugural examination of surgical interventions for RD/RB and MH secondary to OT in the literature. Within our meta-analysis, we delineate the study selection and characteristics, encompassing 14 studies that were incorporated into the review, with a collective analysis of 97 eyes. The findings indicate a notable success rate in achieving retinal reattachment of RD/RB and closure of MHs through surgical management. Furthermore, BCVA demonstrated improvement in both RD/RB and MH analyses post-surgery. Importantly, the overall rates of general complications were significantly reduced.

OT is characterized by self-limiting necrotizing retinochoroiditis, vitritis, and inflammation in the anterior segment. Active OT is characterized by focal necrotizing retinochoroiditis that is usually unilateral and may be congenital or acquired [13, 26–29]. RD and RB, a leading cause of blindness in ocular toxoplasmosis, results from posterior vitreous detachment due to intraocular inflammation, requiring surgical treatment [14, 30].

Earlier investigations have identified various risk factors associated with RD and RB. These factors encompass age, myopia, severe inflammation, positive family history, as well as a history of trauma, cataract surgery, and diabetes [14, 15, 31]. In this meta-analysis, the risk factors identified were myopia and proliferative vitreoretinopathy (Table 1).

Furthermore, as shown in Table 2, the clinical treatment for OT typically involves antimicrobial medications, with or without the addition of corticosteroids in oral, topical, or intravitreal administration. Various drugs have been suggested, including pyrimethamine, sulfadiazine, spiramycin, clindamycin, and trimethoprim-sulfamethoxazole. The use of corticosteroids in ocular toxoplasmosis remains a topic of debate; these medications are primarily employed to mitigate severe inflammatory reactions. However, it is important to note that corticosteroids in OT may potentially impact the development of vitreoretinal traction and preretinal membranes [18, 32]. This influence may contribute to the development of RD or RB.

The analysis revealed that retinal reattachment was successful in 97% of the patients with RD or RB and 96% of the patients with just RD, indicating the effectiveness of the treatments used to address RD and RB associated with OT. Simple operations would demonstrate better outcomes in cases of RD and RB, while combined procedures that were associated with higher risk of poor results could be indicated for more complex cases of RD with risk factors [12]. Prior studies have explored diverse surgical approaches. In the analysis, PPV and/or SB in conjunction with silicon oil infusion or gas infusion were the predominant techniques (Table 1).

Within 1 year of experiencing retinochoroiditis, according to the analysis, approximately 62% of patients develop retinal detachment or breaks. This happens because of *T. gondii*'s ability to access the human retina through various routes, including leukocyte taxis, transmigrating tachyzoites, and infecting endothelial cells [13, 33]. Once within the retina, these tachyzoites can traverse retinal layers and access various retinal host cells [27, 33].

RD is not a common occurrence in OTs, as evidenced by a study conducted in the Netherlands that involved 154 patients and reported a frequency of approximately 6% [28]. However, a meta-analysis has shown that there is a recurrence rate of 14% for retinal detachment after reattachment surgery. Recurrent retinochoroiditis is prevalent in OT, as evidenced by a substantial retrospective case series indicating recurrences in 79% of patients monitored for an average of > 5 years [28, 34]. This underscores a potential association with recurrent RD. These findings emphasize the importance of long-term monitoring and further research to develop interventions that can effectively minimize the likelihood of recurrent detachment in these patients.

The literature suggests that ocular toxoplasmosis often affects the macula, leading to a significant decline in visual acuity. However, the present study showed that surgical treatment can substantially improve BCVA, with an average increase of 0.60 LogMAR. The factors such as patient-specific risk profiles and the surgeon's expertise with different techniques play crucial roles in determining the optimal improvement in BCVA. These considerations guide the selection of surgical treatments in such cases. Evidence supporting this comes from a prior study, where patients retaining their visual potential were found to have attached RBs. This correlation could be explained by the detection of these RBs during ophthalmologic examinations conducted as part of active OT attacks, leading to early intervention and treatment [14, 29].

The overall complication rate of ocular toxoplasmosis has been reported to be 25%. The primary reported complication is postoperative glaucoma or elevated IOP. This observation may be associated with a greater number of intraocular surgeries performed [12, 15]. However, further investigations of the specific nature and clinical significance of these complications are essential to gain a more comprehensive understanding of the challenges faced by individuals undergoing treatment and recovering from ocular toxoplasmosis [33, 35].

As depicted in Table 2, PPV with internal limiting membrane peeling is considered a potentially effective treatment for MHs in the context of uveitis [36]. This approach aims to relieve forces contributing to MH development [16]. Despite limited published literature, MH associated with uveitis often involves a more fragile retina due to prior inflammation, potentially influencing functional outcomes. This meta-analysis demonstrated a high success rate in MH closure achieved in 98% of patients. BCVA improved by a mean difference of 0.67 after surgeries, indicating the effectiveness of treatments for MH secondary to OT. Sousa et al. propose that the success rate may be attributed to factors such as patient selection and the surgical procedure. Specifically, patients with MH characterized by elevated borders and a visibly thick nonatrophic fovea had a higher probability of closure and improved vision potential following PPV and internal limiting membrane peeling. Additionally, the limited occurrence of large macular holes may have contributed to the overall success rate. Another favorable factor was the relatively short period between symptom onset and surgery [16].

### Limitations

Although the results yielded significant insights into the safety and efficacy of surgical management for RD/RB and MH secondary to OT, it is crucial to take into consideration the limitations of this study. These studies were limited by several factors, including their retrospective nature and the variability of baseline patient features and treatment regimens. There are a relatively small number of patients, and the different types of RD and MH varies greatly among studies, the RD/RB and MH severity may be more advanced in some patients than another, potentially affecting the baseline characteristics. Other limitation of the present studies was the absence of information on various risk factors RD/RB and MH that could have confounded the severity of patients' prognoses. Many studies included in this analysis reported follow-up periods ≥ 1 month. However, long-term outcomes (≥ 12 months) beyond the follow-up period were assessed in just a few studies. It is crucial to assess the long-term stability of surgical effects.

## Conclusions

In conclusion, this meta-analysis provides significant insights into the management RD/RB and MH secondary to OT. The study showed that the treatment approaches currently in use are effective, with a remarkable rate of retinal reattachment, MH closure and substantial improvements in visual acuity. However, the risk of complications and recurrence of RD highlights the importance of continuing research and refining treatment strategies to enhance the overall quality of care for people affected by ocular toxoplasmosis. Further randomized and long-term studies with evaluation of factors related to the disease and its surgical treatment and their influence on both anatomical and visual outcomes could provide valuable insights.

## Data Availability

The datasets used and analyzed during the current study are available from the corresponding author upon reasonable request.

## Abbreviations

RD: Retinal detachment
RPE: Retinal pigment epithelium
RB: Retinal break
SB: Scleral buckling
PPV: Pars plana vitrectomy
PR: Pneumatic retinopexy
OT: Ocular toxoplasmosis
RR: Relative risk
MD: Mean difference
IPDMA: Individual patient data meta-analysis
BCVA: Best-corrected visual acuity

## References

[1] Delair E, Latkany P, Noble AG, Rabiah P, McLeod R, Brézin A (2011). Clinical manifestations of ocular toxoplasmosis. Ocul Immunol Inflamm.

[2] Atmaca LS, Simsek T, Batioglu F (2004). Clinical features and prognosis in ocular toxoplasmosis. Jpn J Ophthalmol.

[3] Jasper S, Vedula SS, John SS, Horo S, Sepah YJ, Nguyen QD (2017). Corticosteroids as adjuvant therapy for ocular toxoplasmosis. Cochrane Database Syst Rev.

[4] Song HB, Jung BK, Kim JH, Lee YH, Choi MH (2018). Investigation of tissue cysts in the retina in a mouse model of ocular toxoplasmosis: distribution and interaction with glial cells. Parasitol Res.

[5] Zamora DO, Rosenbaum JT, Smith JR (2008). Invasion of human retinal vascular endothelial cells by Toxoplasma gondii tachyzoites. Br J Ophthalmol.

[6] Vallochi AL, Nakamura MV, Schlesinger D, Martins MC, Silveira C, Belfort R (2002). Ocular toxoplasmosis: more than just what meets the eye. Scand J Immunol.

[7] Holland GN (2003). Ocular toxoplasmosis: a global reassessment. Part I: epidemiology and course of disease. Am J Ophthalmol.

[8] Ghazi NG, Green WR (2002). Pathology and pathogenesis of retinal detachment. Eye (Lond).

[9] Znaor L, Medic A, Binder S, Vucinovic A, Marin Lovric J, Puljak L (2019). Pars plana vitrectomy versus scleral buckling for repairing simple rhegmatogenous retinal detachments. Cochrane Database Syst Rev.

[10] Hillier RJ, Felfeli T, Berger AR, Wong DT, Altomare F, Dai D (2019). The pneumatic retinopexy versus vitrectomy for the management of primary rhegmatogenous retinal detachment outcomes randomized trial (PIVOT). Ophthalmology.

[11] Page MJ, McKenzie JE, Bossuyt PM, Boutron I, Hoffmann TC, Mulrow CD (2021). The PRISMA 2020 statement: an updated guideline for reporting systematic reviews. BMJ.

[12] Moreira FV, Iwanusk AM, Amaral ARD, Nóbrega MJ, Novelli FJ (2018). Surgical outcomes of rhegmatogenous retinal detachment associated with ocular toxoplasmosis. Arq Bras Oftalmol.

[13] Faridi A, Yeh S, Suhler EB, Smith JR, Flaxel CJ (2015). Retinal detachment associated with ocular toxoplasmosis. Retina.

[14] Bosch-Driessen LH, Karimi S, Stilma JS, Rothova A (2000). Retinal detachment in ocular toxoplasmosis. Ophthalmology.

[15] Adan A, Giralt J, Alvarez G, Alforja S, Burés-Jesltrup A, Casaroli-Marano RP (2009). Pars plana vitrectomy for vitreoretinal complications of ocular toxoplasmosis. Eur J Ophthalmol.

[16] Sousa DC, Andrade GC, Nascimento H, Maia A, Muccioli C (2021). Macular hole associated with toxoplasmosis: a surgical case series. Retin Cases Brief Rep.

[17] Lucena D, Ribeiro A, Lucena D, Lucena A, Jorge R (2009). Roturas retinianas em retinocoroidite por toxoplasmose: série de casos. Arq Bras De Oftalmol.

[18] Kianersi F, NaderiBeni A, Ghanbari H, Fazel F (2012). Ocular toxoplasmosis and retinal detachment: five case reports. Eur Rev Med Pharmacol Sci.

[19] Caplan H, Wakabayashi T, Klufas M, Mehta S, Deaner J, Dunn JP (2023). Prevalence, characteristics, and outcomes of retinal detachment associated with toxoplasmosis retinochoroiditis. Investigat Ophthalmol Visual Sci.

[20] Erol MK, Bozdogan YC, Suren E, Gedik B (2022). Treatment of a full-thickness macular hole and retinal detachment secondary to toxoplasma chorioretinitis that developed shortly after COVID-19: a case report. J Fr Ophtalmol.

[21] Tanaka R, Obata R, Sawamura H, Ohtomo K, Kaburaki T (2014). Temporal changes in a giant macular hole formed secondary to toxoplasmic retinochoroiditis. Can J Ophthalmol.

[22] Arana B, Fonollosa A, Artaraz J, Martinez-Berriotxoa A, Martinez-Alday N (2014). Macular hole secondary to toxoplasmic retinochoroiditis. Int Ophthalmol.

[23] Ikeda M, Baba T, Aikawa Y, Yotsukura J, Yokouchi H, Yamamoto S (2021). Case of macular hole secondary to ocular toxoplasmosis treated successfully by vitrectomy with inverted internal limiting membrane flap. Case Rep Ophthalmol.

[24] Doshi S, Gulati M, Pathengay A, Hegde S (2020). Spontaneous closure of macular hole in a case of toxoplasma retinochoroiditis. Indian J Ophthalmol.

[25] Scott NL, Sridhar J, Flynn HW (2018). Management of giant retinal tear and retinal detachment in a patient with active toxoplasmosis retinochoroiditis. Am J Ophthalmol Case Rep.

[26] Dodds EM, Holland GN, Stanford MR, Yu F, Siu WO, Shah KH (2008). Intraocular inflammation associated with ocular toxoplasmosis: relationships at initial examination. Am J Ophthalmol.

[27] London NJ, Hovakimyan A, Cubillan LD, Siverio CD, Cunningham ET (2011). Prevalence, clinical characteristics, and causes of vision loss in patients with ocular toxoplasmosis. Eur J Ophthalmol.

[28] Bosch-Driessen LE, Berendschot TT, Ongkosuwito JV, Rothova A (2002). Ocular toxoplasmosis: clinical features and prognosis of 154 patients. Ophthalmology.

[29] Holland GN (2004). Ocular toxoplasmosis: a global reassessment. Part II: disease manifestations and management. Am J Ophthalmol.

[30] Iwahashi-Shima C, Sato T, Bando H, Ikeda T, Emi K (2013). Anatomic and functional outcomes of 25-gauge vitrectomy for repair of eyes with rhegmatogenous retinal detachment complicated by proliferative vitreoretinopathy. Clin Ophthalmol.

[31] Kovačević-Pavićević D, Radosavljević A, Ilić A, Kovačević I, Djurković-Djaković O (2012). Clinical pattern of ocular toxoplasmosis treated in a referral centre in Serbia. Eye (Lond).

[32] Holland GN, Lewis KG (2002). An update on current practices in the management of ocular toxoplasmosis. Am J Ophthalmol.

[33] Smith JR, Ashander LM, Arruda SL, Cordeiro CA, Lie S, Rochet E (2021). Pathogenesis of ocular toxoplasmosis. Prog Retin Eye Res.

[34] Englander M, Young LH (2011). Ocular toxoplasmosis: advances in detection and treatment. Int Ophthalmol Clin.

[35] Weiss LM, Dubey JP (2009). Toxoplasmosis: a history of clinical observations. Int J Parasitol.

[36] Mizuno M, Fujinami K, Watanabe K, Akiyama K (2015). Macular hole associated with Vogt-Koyanagi-harada disease at the acute Uveitic stage. Case Rep Ophthalmol.

